# Widespread Secondary Contact and New Glacial Refugia in the Halophilic Rotifer *Brachionus plicatilis* in the Iberian Peninsula

**DOI:** 10.1371/journal.pone.0020986

**Published:** 2011-06-16

**Authors:** Sergi Campillo, Manuel Serra, María José Carmona, Africa Gómez

**Affiliations:** 1 Institut Cavanilles de Biodiversitat i Biologia Evolutiva, Universitat de València, Valencia, Spain; 2 Department of Biological Sciences, University of Hull, Hull, United Kingdom; Field Museum of Natural History, United States of America

## Abstract

Small aquatic organisms harbour deep phylogeographic patterns and highly structured populations even at local scales. These patterns indicate restricted gene flow, despite these organisms' high dispersal abilities, and have been explained by a combination of (1) strong founder effects due to rapidly growing populations and very large population sizes, and (2) the development of diapausing egg banks and local adaptation, resulting in low effective gene flow, what is known as the Monopolization hypothesis. In this study, we build up on our understanding of the mitochondrial phylogeography of the halophilic rotifer *Brachionus plicatilis* in the Iberian Peninsula by both increasing the number of sampled ponds in areas where secondary contact is likely and doubling sample sizes. We analyzed partial mitochondrial sequences of 252 individuals. We found two deep mitochondrial DNA lineages differing in both their genetic diversity and the complexity of their phylogeographic structure. Our analyses suggest that several events of secondary contact between clades occurred after their expansion from glacial refugia. We found a pattern of isolation-by-distance, which we interpret as being the result of historical colonization events. We propose the existence of at least one glacial refugium in the SE of the Iberian Peninsula. Our findings challenge predictions of the Monopolization hypothesis, since coexistence (i.e., secondary contact) of divergent lineages in some ponds in the Iberian Peninsula is common. Our results indicate that phylogeographic structures in small organisms can be very complex and that gene flow between diverse lineages after population establishment can indeed occur.

## Introduction

Essential to understand the evolution of organic diversity, phylogeography studies the principles and processes that govern the geographical distribution of genetic lineages, mainly at the within species level [1], [2]. Although Avise gave zooplankton dwelling in ponds and lakes a fleeting treatment in his topical book [1], a consensus has built up in the last decade pointing to the existence of deep phylogeographic structures in these organisms [3]–[7]. This consensus strongly contrasts with the traditional view that zooplanktonic organisms should have high rates of gene flow due to high dispersal abilities; see for instance [8]. This contrast is known as the “dispersal-gene flow” paradox [4], since high gene flow should hamper the formation of deep phylogeographic structures.

The Monopolization hypothesis [4] was proposed to solve the paradox. The hypothesis extends the explanation of Boileau et al. [9] and states that gene flow after colonization should be strongly restricted due to a combination of factors. After the colonization of a habitat, the offspring of the population founders rapidly monopolizes the habitat, effectively hampering the success of later immigrants due to two factors: (1) their high population growth rates, building up extremely large populations relatively quickly, and (2) their rapid adaptation to local conditions. Therefore, a prediction of the hypothesis would be the occurrence of sharp contact zones between intraspecific phylogeographic lineages, with little or no overlap between them. In contrast, studies on the cladoceran *Daphnia magna* and on the rotifer *Brachionus manjavacas* have revealed signatures of secondary contact, e.g. [3], [6]. Following De Gelas and De Meester [3], under the monopolisation hypothesis model, there is a small but non-zero probability that population founders will belong to different phylogenetic groups than the regional one – due to long distance dispersal likely to be mediated by birds [10]. Nevertheless, if secondary contact is a general pattern in continental zooplankton, it could constitute a challenge for the hypothesis.

The Iberian Peninsula has been recognized as one of the major European glacial refugia during the Pleistocene glaciations [11]. Instead of forming a single, homogeneous glacial refugium, however, its complex orography and climatic diversity allowed the isolation of populations in separated areas during glacial maxima [12] resulting in patterns of phylogeographic concordance among different species which survived in the Peninsula during glaciations. For example, for terrestrial fauna at least seven putative glacial refugia were recognized while for freshwater fish glacial refugia coincided with the main river basins [12].

**Figure 1:**
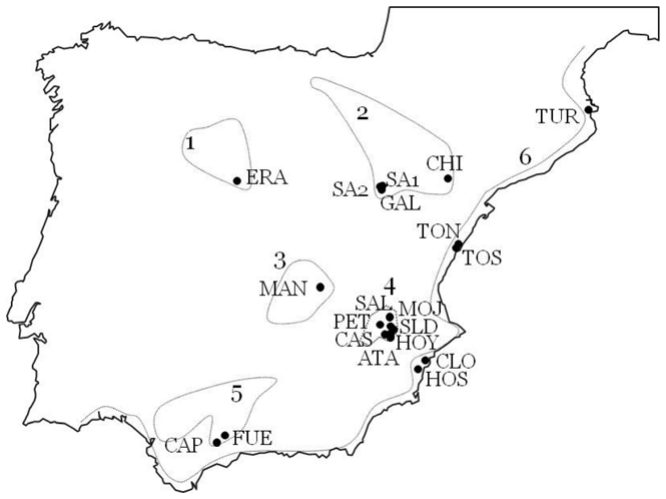
Map of the salt lake areas in the Iberian Peninsula showing the sampled *B. plicatilis* populations. See pond acronyms in Table 1. Basins: 1, Duero; 2, Ebro; 3, Guadiana; 4, Júcar-Segura; 5, Guadalquivir; 6, coastal lagoons.

Iberian saline environments hold assemblages of relatively specialized fauna [13]. The distribution of saline environments in several drainage basins (see Figure 1) and a coastal chain of lagoons makes the establishment of concordance hypothesis feasible. In the last few years, new studies on the phylogeography of Iberian halophilic invertebrates allow for the evaluation of concordance patterns in these systems [5]–[7], [14], [15]. Some of these studies have been performed in the rotifer species complex *Brachionus plicatilis*, a group comprising approximately 12 cryptic species inhabiting coastal marine environments and salt lakes worldwide [16], [17]. Strong concordance between phylogeographic patterns of two ecologically similar species belonging to this complex, *B. plicatilis* and *B. manjavacas*, has been found in the Iberian Peninsula. Both species show two deep mitochondrial DNA lineages with a geographic orientation independent of the coastal/inland dichotomy [5], [6]. One of these lineages is more diverse and has a strong phylogeographical structure, with a remarkable similarity between the phylogeographic patterns of these species-specific lineages. In addition, *B. manjavacas* shows widespread secondary contact between mtDNA lineages, and indications of secondary contact have also been found in *B. plicatilis* [5], [18].

Obtaining reliable phylogeographic results is sensitive to the spatial sampling scope. In order to assess previous conclusions and to gain a better understanding of phylogeography in the Iberian Peninsula, we investigate *B. plicatilis* mtDNA variation in areas where secondary contact is suspected, and increase sample sizes in other habitats in the Iberian Peninsula to double the number of *B. plicatilis* sequences. Specifically, we want to ascertain the extent of secondary contact in this species, drawing conclusions on the processes involved and the implications for the Monopolization hypothesis. Finally, we review and discuss concordance patterns between the phylogeographies of *B. plicatilis*, and other organisms inhabiting Iberian halophilic habitats.

## Materials and Methods

Rotifers of the species complex *Brachionus plicatilis* combine both sexual and asexual reproduction, i.e. are cyclical parthenogens, e.g. [15]. In a typical growth cycle, the active population is initiated by hatching of diapausing eggs from the sediment of the pond or lake. These hatchlings are diploid asexual females produce genetically identical daughters by parthenogenesis. After a period of clonal propagation, a sexual phase starts when, induced by environmental factors, such as population density, asexual females start produce sexual females among their offspring [19]–[23]. Sexual females produce haploid eggs. If a sexual female is not inseminated in the first hours of her life, her unfertilized eggs will develop into haploid eggs [24]. If inseminated, her fertilized, sexually produced, eggs will result in a diploid embryos, the so-called diapausing eggs. All members of the *B. plicatilis* species complex investigated to date show this cyclical parthenogenetic life cycle.

We isolated *B. plicatilis* diapausing eggs from sediment samples from six Spanish ponds using a sugar flotation technique [25]. We extracted DNA from diapausing eggs using Chelex [26], [27]. In order to select diapausing eggs belonging to *B. plicatilis* from the morphologically similar ones from other syntopic species of the *B. plicatilis* cryptic species complex, we either used a barcoding approach [28] and sequenced directly polymerase chain reaction (PCR) products (a fragment of the COI mitochondrial gene, see below) in order to use only *B. plicatilis* sequences, or conducted analysis by restriction fragment length polymorphism (RFLP) on PCR amplifications of the same gene [26] prior to sequencing. The latter approach was used in those lakes where *B. plicatilis* was not the dominant species in the diapausing egg bank.

We used specific primers COIdgF and COIdgR –see [17] for details– to amplify a fragment of the mitochondrial gene cytochrome *c* oxidase subunit 1 (cox1 or COI). We performed PCR in a total of 50 µl containing 3 µl of template DNA, 0.2 mM of each nucleotide, 0.6 µM of each primer, 1.5 U of *Taq* polymerase (BIOTOOLS) and 1× BIOTOOLS buffer (producing a final concentration of 2 mM MgCl_2_). A Mastercycler™ (Eppendorf) was used for PCR amplification with a cycling profile of 3 min at 94°C; 40 cycles of 30 s at 94°C, 1 min at 48°C and 1 min at 72°C; and a final extension step of 7 min at 72°C. PCR were purified (High Pure PCR Product Purification Kit, Roche) and sequenced using the ABI PRISM Dye Terminator Cycle Sequencing Ready Reaction Kit (Perkin-Elmer) on an ABI 3700 automated sequencer.

We computed the number of haplotypes per population and haplotype diversity (*Hd*) using DnaSP version 4.50.1 [29]. We performed a rarefaction analysis using RAREFAC (available from R. Petit at http://www.pierroton.inra.fr/genetics/labo/Software/Rarefac/index.html) to calculate standardized allelic richness (*A*) for each sampled population. We set the rarefaction size to 5. Thus, seven populations with *n*<5 (Poza Norte de Torreblanca, Mojón Blanco, Fuente de Piedra, El Saladar, Clot de Galvany, Atalaya de los Ojicos and Casa Nueva) were excluded from this analysis.

We calculated pairwise estimates of population differentiation (*F*_ST_) using Arlequin 3.1 (30) applying the available evolutionary model closest to that recommended by ModelGenerator (see below). We performed a reduced major axis (RMA) linear regression between *F*_ST_ estimates and geographical distances. As the regression contained multiple pairwise comparisons, the Mantel test for significance was employed with 30,000 randomizations. All regression related analyses were carried out in IBDWS v 3.15 [31].

We constructed an alignment using the program MEGA5 [32] with all the individual sequences. Identical sequences were then collapsed using Collapse v. 1.2 [33]. We constructed a maximum likelihood phylogenetic tree of all haplotypes using the program MEGA5 [32] with 1000 pseudorreplicates to assess branch robustness, and the best fit evolutionary model to our dataset according to ModelGenerator v. 0.84 [34]. We used two outgroups: one from Canada (GenBank AF499055.1) and the other from Australia (GenBank AF524543) both from the species *B. plicatilis*.

We used Nested Clade Phylogeographic Analysis (NCPA) [35] to test for associations between geography and genealogy. This method allows distinguishing between contemporary and historical events (for example, gene flow from allopatric fragmentation). We followed the rules by Templeton [36] to construct the nested design on the network using the TCS v1.18 program [37]. This version of the program does not include the following nesting rule that we applied to construct the nested design: “When the nesting rules at level *n* create an interior, degenerate *n*-step clade [an *n*-step clade with only (*n*−1)-step member with actual observations, the other (*n*−1)-step member or members being interior nodes without any observations], such clades are nested at the (*n*+1) level with the *n*-step clade that is mutationally closest to the one (*n*−1)-step member of the degenerate clade with observations” (D. Posada and A.R. Templeton, personal communication). The program GeoDis 2.6 [38] was used to compute the distance measures: the clade distance (*D_c_*), which measures the geographical range of a particular clade, and the nested-clade distance (*D_n_*), which measures how a particular clade is geographically distributed relative to its closest evolutionary sister clade (i.e. clades in the same higher-step nesting category) [27]. GeoDis also computes the statistical significance of these distance measures by a Chi-square test after 10,000 random permutations of clades against sampling locality. This version of the program includes a Dunn-Šidák correction for multiple tests.

We then applied the inference key (version 28^th^ April 2009) establishing the phylogeographic inferences for each clade. To detect secondary contact, we performed the analysis suggested in Templeton [39] and previously applied to the *B. manjavacas* Iberian phylogeography [6]. In those higher-level clades with signs of population expansion inferred by the NCPA, we performed a mismatch distribution analysis [40] in order to independently test the demographic expansion by using DNAsp version 4.50.1 [29].

## Results

We sequenced a total of 154 individuals from the six sampled populations (GenBank accession numbers FJ387332–FJ387485), to which we added the 98 previously sequenced for Iberian populations (GenBank accession numbers: AF266853–AF266950) [5]. *B. plicatilis* was found in 20 ponds in the Iberian Peninsula, in the following basins: Duero, Ebro, Guadiana, Júcar-Segura, Guadalquivir, and in five ponds of the coastal Mediterranean chain (Figure 1). The 252 *B. plicatilis* cytochrome oxidase *c* subunit I (COI) sequences collapsed in a total of 29 haplotypes (Table 1). On average, 12.60 individuals were sequenced per population (range: 2–36; see also Figure S1). The alignment included 581 bp with no insertions, deletions, stop codons or ambiguities. There were 49 variable sites (47 synonymous and 2 non-synonymous) and 40 parsimony-informative sites. Since this alignment was slightly shorter than in Gómez et al. [5], haplotypes 18 and 16 from that work merged into one (new haplotype 16).

**Table 1:** Sites from which *Brachionus plicatilis* samples were obtained, number of individuals sequenced (*N*) and source of the data.

Site	Acronym	Location	*N*	*A*	*Hd*	(SD)	Haplotypes (number of individuals)
Atalaya de los Ojicos	ATA	38°46′22″N, 1°25′50″W	3	-	-		H21 (3)
Balsa de Santed 1	SA1	41°00′59″N, 1°32′31″W	23*	1.150	0.55	(0.06)	H13 (9), H16 (13), H27 (1)
Balsa de Santed 2	SA2	41°01′07″N, 1°32′36″W	10	0.000	0.00	(0.00)	H16 (10)
Capacete	CAP	37°01′21″N, 4°49′35″W	5	0.000	0.00	(0.00)	H16 (5)
Casa Nueva	CAS	38°46′45″N, 1°26′08″W	2	-	-		H21 (2)
Clot de Galvany	CLO	38°15′0″N, 0°32′13″W	2	-	-		H21 (2)
El Saladar	SLD	38°47′16″N, 1°25′09″W	4	-	-		H20 (3), H21 (1)
Estany d'en Túries	TUR	42°14′08″N, 3°06′50″E	7	2.381	0.81	(0.13)	H1 (3), H2 (2), H4 (1), H6 (1)
Fuente de Piedra	FUE	37°06′38″N, 4°46′07″W	2	-	-		H17 (2)
Gallocanta	GAL	40°58′12″N, 1°30′00″W	6	0.000	0.00	(0.00)	H13 (6)
Hondo Sur	HOS	38°10′04″N, 0°43′34″W	30*	1.755	0.66	(0.07)	H16 (1), H22 (16), H23 (8), H24 (2), H25 (2), H26 (1)
Hoya Rasa	HOY	38°47′05″N, 1°25′39″W	27*+2	0.900	0.47	(0.06)	H20 (10), H21 (19)
Las Eras	ERA	41°12′04″N, 4°34′56″W	10	0.500	0.20	(0.15)	H14 (9), H15 (1)
Manjavacas	MAN	39°25′03″N, 2°51′43″W	9	0.000	0.00	(0.00)	H19 (9)
Mojón Blanco	MOJ	38°47′53″N, 1°25′59″W	2	-	-		H16 (1), H21 (1)
Pétrola	PET	38°50′28″N, 1°33′54″W	9	0.833	0.39	(0.16)	H20 (2), H21 (7)
Poza Norte de Torreblanca	TON	40°08′55″N, 0°10′08″E	2	-	-		H3 (1), H10 (1)
Poza Sur de Torreblanca	TOS	40°08′42″N, 0°10′03″E	26*+10	1.035	0.60	(0.08)	H5 (1), H7 (1), H8 (22), H9 (6), H28 (1), H29 (4), H30 (1)
Salada de Chiprana	CHI	41°14′22″N, 0°10′57″W	28*+8	0.278	0.11	(0.07)	H11 (34), H12 (1), H13 (1)
Salobrejo	SAL	38°54′50″N, 1°28′06″W	20*+5	0.000	0.00	(0.00)	H21 (25)

*Sequences obtained in this study.

Standardized allelic richness (*A*) and number of individuals per haplotype found in each pond for *Brachionus plicatilis*. *Hd* is the haplotype diversity (standard deviation within parentheses), calculated only when five or more individuals were sequenced.

Twenty-five out of the 29 haplotypes were found in single ponds. The most widespread haplotype (number: 21) was found in eight ponds, seven of them from the Júcar-Segura basin, and the remaining (Clot de Galvany) from the coastal chain, but close to the former basin. There was an average of 2.2 haplotypes per pond. Among the populations showing the highest standardized allelic-richness (*A*), three were coastal (Estany d'en Túries, Hondo Sur and Poza Sur de Torreblanca) and one inland (Balsa de Santed 1). Standardized allelic richness for ponds with more than 5 individuals sequenced ranged from 0 to 2.831 (Table 1).

**Figure 2:**
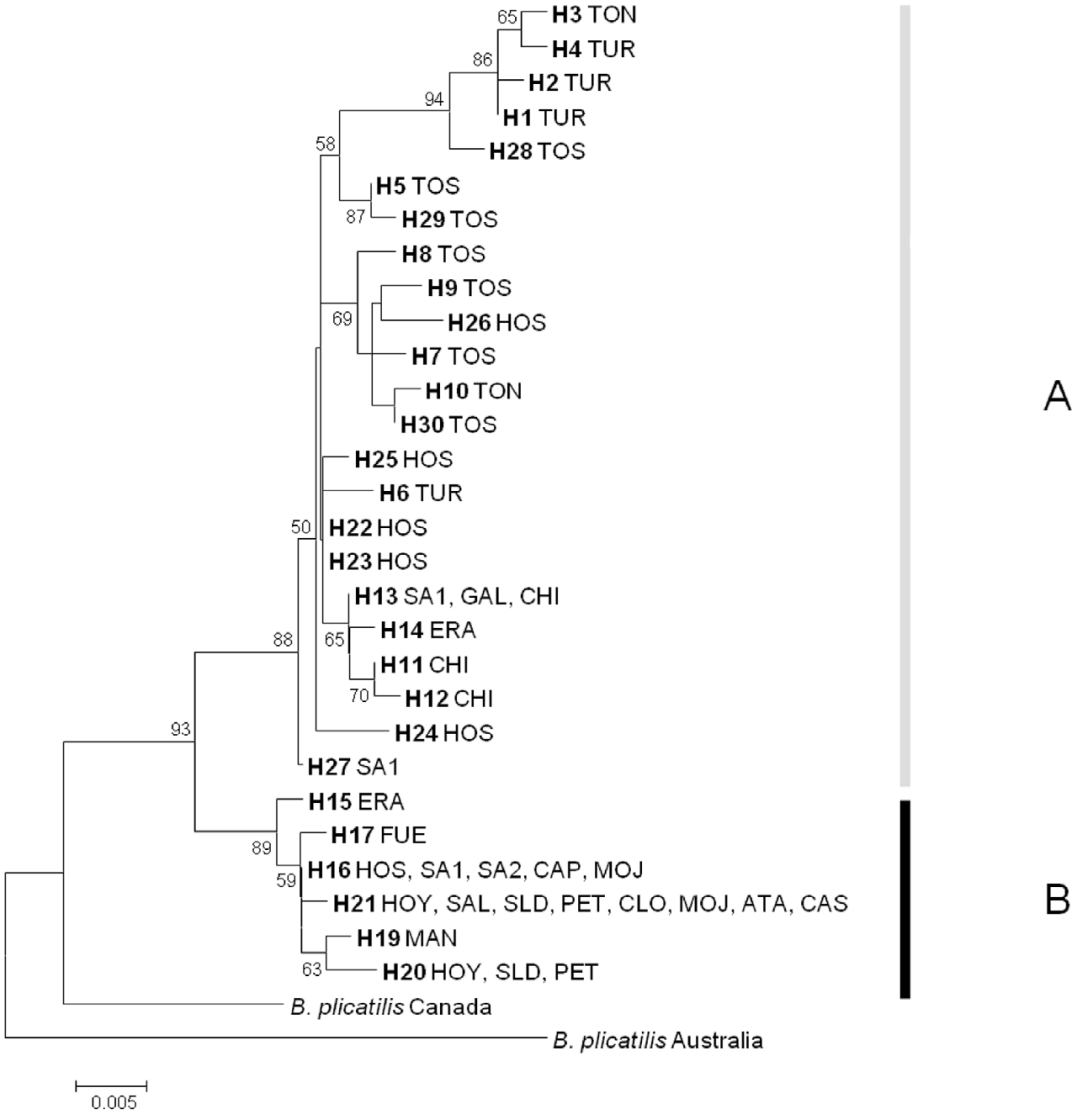
Midpoint rooted maximum likelihood phylogram of *B. plicatilis* mtDNA haplotypes. Percent bootstrap support, if >50%, is shown. See Table 1 for pond acronyms.

The Akaike Information Criterium II and Bayesian Information Criterium implemented in ModelGenerator v. 0.84 selected the Hasegawa-Kishino-Yano plus Gamma model (HKY+G; G = 0.15) as the best fit model of evolution for our dataset [41], with base frequencies of A = 0.20, C = 0.22, G = 0.20, and T = 0.38. The phylogenetic tree (Figure 2) showed two strongly supported Iberian lineages: lineage A, which contains predominantly northern haplotypes (inland and coastal), plus Hondo Sur, a south-eastern coastal lagoon; and lineage B, which contains predominantly southern inland haplotypes with presence also in two ponds from the coastal chain (Clot de Galvany and Hondo Sur) and some northern inland ponds (Las Eras, Balsa de Santed 1 and Balsa de Santed 2). Lineages A and B were found in 8 and 14 ponds, respectively and co-occurred in three ponds, with frequencies of lineage A in the range 3–57%. Both lineages differed in their haplotype diversity, lineage A with 23 haplotypes and lineage B with 6. The average uncorrected *p*-distance between these lineages is 2.7%.

**Figure 3:**
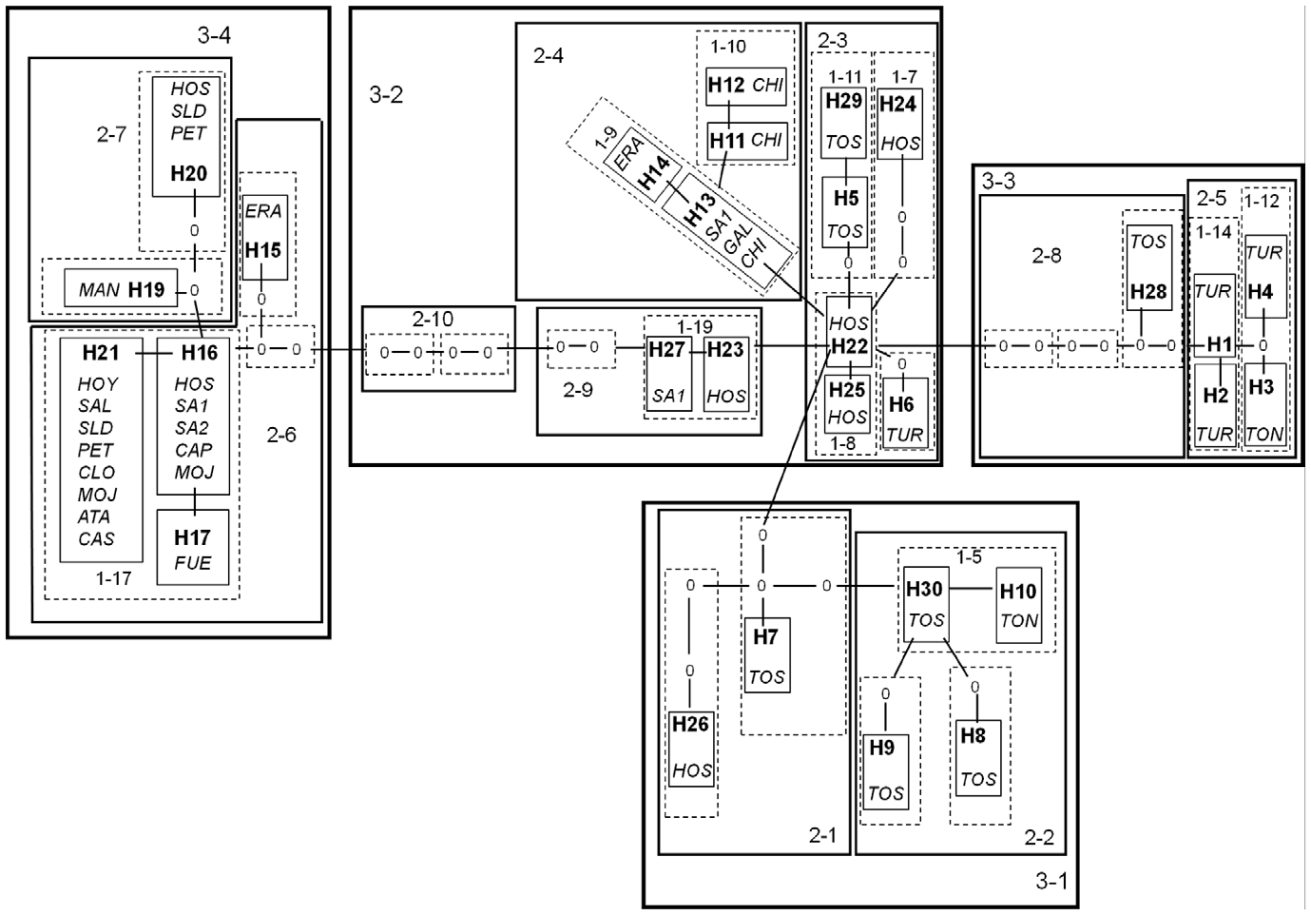
Statistical parsimony cladogram and nested design for the 29 cytochrome oxidase haplotypes found in *B. plicatilis*. We designated haplotypes by an H followed by a number; zeros represent missing intermediate haplotypes. We refer to clades as *x*-*y*, where *x* represents the nesting step (from the haplotype step to the 3^rd^ step) and *y* represents the number assigned to that particular clade. Each line represents one mutation. All connections indicated by solid line between haplotypes are supported to be parsimonious at the 95% level. Locations of origin for each haplotype are given in italics.

The haplotype network consisted in 29 haplotypes, 25 1^st^-step clades, 10 2^nd^-step clades and four 3^rd^-step clades (Figure 3). The maximum level of parsimonious steps (*P*>0.95) was 10 and no connection between haplotypes exceeded this number (maximum of 8 steps between haplotypes 27 and 16).

**Figure 4:**
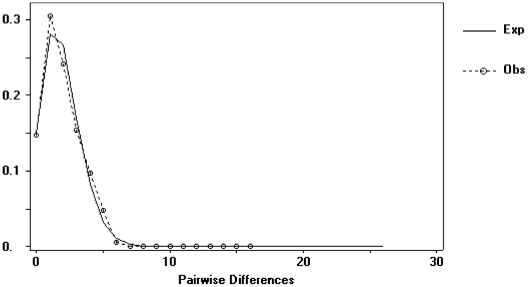
Results of the mismatch distribution for clade 3-2. Solid line represents the number of observed pairwise nucleotide differences among *B. plicatilis* clades within the clade. The dash line represents the expected distribution of nucleotide differences under a model of recent population expansion. Raggedness statistic *r* = 0.0437.

Eight out of 17 clades with geographical and genetic variation resulted in significant permutational contingency tests, indicating non-random geographical distribution of haplotypes (Table 2). The NCPA inferred restricted gene flow / dispersal but with some long distance dispersal in two clades (clade 1–17 and the total cladogram). Clades 2-4 and 3-4 showed signs of restricted gene flow with isolation by distance. Clade 2-3 (found in coastal ponds) showed a contiguous range expansion and clade 3-2 (found in coastal and northern inland ponds) showed signs of long distance colonization, seemingly from southern coastal ponds to northern coastal ones and to those of the Ebro valley. The mismatch analysis for the clade 3-2 (Fig. 4) also supported a recent event of population expansion. Finally, clades 2-7 and 1-9 showed signs of allopatric fragmentation, between Hoya Rasa, El Saladar and Pétrola (Júcar-Segura basin) and Manjavacas (Guadiana basin) on one hand, and between Ebro and Duero basins, on the other.

**Table 2:** Nested Clade Phylogeographic Analysis of the geographical distribution of *Brachionus plicatilis* haplotypes.

Clade		Distribution	Inference key sequence	Biological Inference
1-9:	H13	SA1, GAL, CHI	1-19 NO	Allopatric fragmentation
	H14	Basin 1		
1-17:	H16	SA1, SA2, MOJ, CAP, HOS	1-2-3-5-6-7-8 YES	Restricted gene flow / dispersal but with some long distance dispersal over intermediate areas not occupied by the species; or past gene flow followed by extinction of intermediate populations
	H17	FUE		
	H21	ATA, HOY, CAS, MOJ, PET, SAL, SLD, CLO		
2-3:	1-6	TUR	1-2-11-12 NO	Contiguous range expansion from HOS to TOS and TUR
	1-7	HOS		
	1-8	HOS		
	1-11	TOS		
2-4:	1-9	Basin 1, SA1, GAL, CHI	1-2-3-4 NO	Restricted gene flow with isolation by distance
	1-10	CHI		
2-7:	1-16	HOY, SLD, PET	1-19 NO	Allopatric fragmentation
	1-18	Basin 3		
3-2:	2-3	HOS, TOS, TON, TUR	1-2-11-12-13 YES	Long distance colonization, past larger range coupled with subsequent extinction
	2-4	Basin 1, SA1, GAL, CHI		
	2-9	HOS, SA1		
3-4:	2-6	Basin 1, Basin 4, Basin 5, SA1, SA2, CLO, HOS	1-2-3-4 NO	Restricted gene flow with isolation-by-distance
	2-7	Basin 3, HOY, PET, SLD		
Total:	3-1	HOS, TOS, TON	1-2-3-5-6-7 YES	Restricted gene flow / dispersal but with some long distance dispersal
	3-2	Basin 5, CHI, SA1, GAL, HOS, TOS, TUR		
	3-3	TOS, TON, TUR		
	3-4	Basin 1, 3, 4 and 5, SA1, SA2, HOS, CLO		

We show the eight clades with significant GeoDis results (all clades showed *p*-value<0.0001), haplotype and clade distributions (see Table 1 for pond acronyms). When we found a clade in all ponds in a basin we only refer the basin number (Figure 1). We show the sequence followed in the inference key (version 28^th^ April 2009). See Figure 3 for the haplotype network and nested design with haplotype and clade designations.

**Figure 5:**
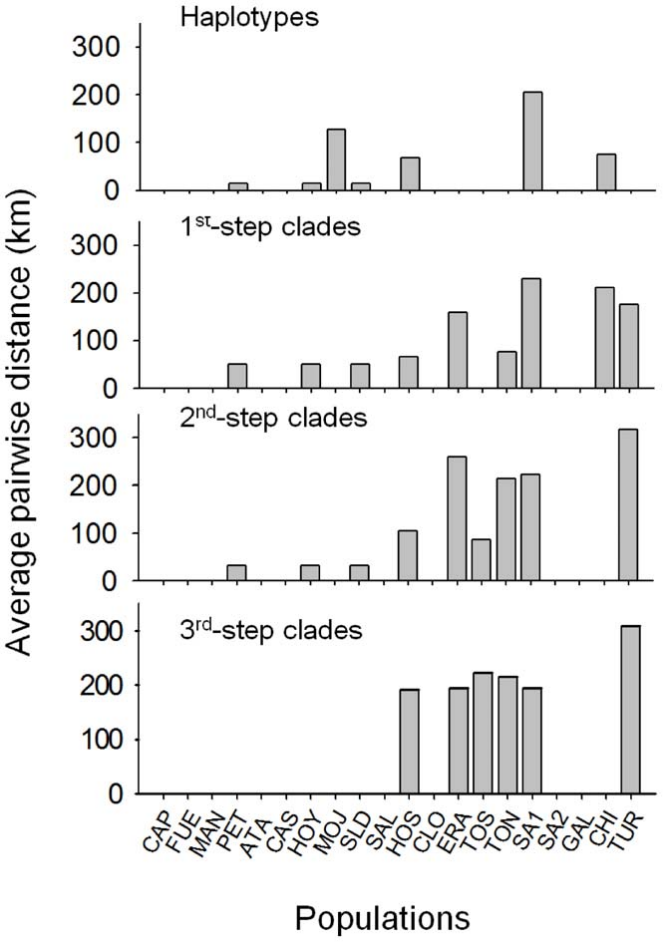
Secondary contact analysis *B. plicatilis*. Ponds where the species was found are ordered on the *x* axis in increasing distances from the most westerly pond to the most easterly. The bars show the average pairwise geographical distance between the geographical centres of haplotypes and clades found at each of the ponds calculated from the coordinates for haplotypes and clades given in GeoDis output. For pond acronyms, see Table 1.

**Figure 6:**
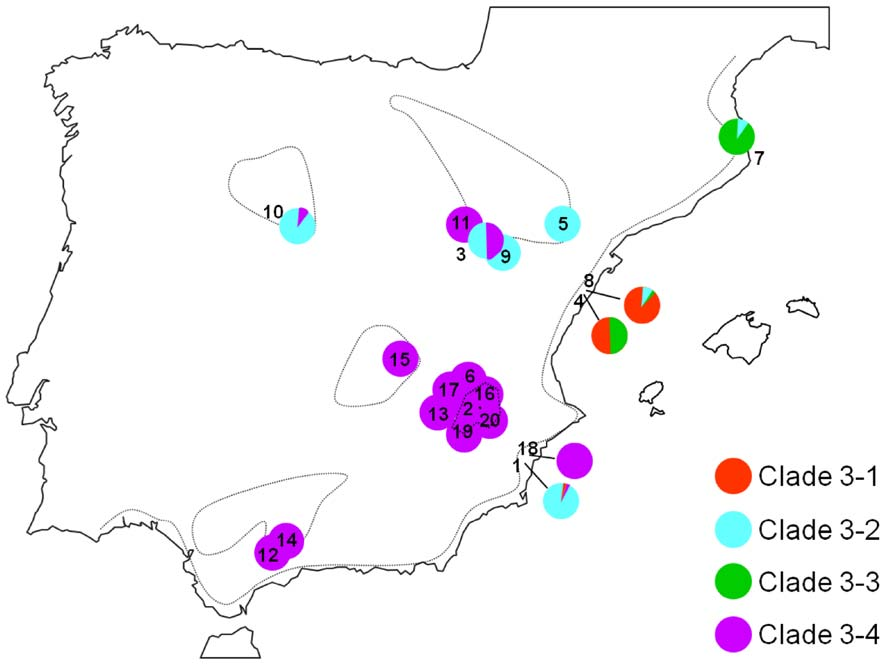
Distribution of 3^rd^-step clades (3-1, 3-2, 3-3 and 3-4; see **Figure 3**) of *B. plicatilis* in the Iberian Peninsula. Note that these highest-step clades are sympatric at six ponds, a signature for secondary contact. 1: HOS; 2: HOY; 3: SA1; 4: TOS; 5: CHI; 6: SAL; 7: TUR; 8: TON; 9: GAL; 10: ERA; 11: SA2; 12: CAP; 13: MOJ; 14: FUE; 15: MAN; 16: SLD; 17: PET; 18: CLO; 19: ATA; 20: CAS. For pond acronyms, see Table 1.

In order to test for secondary contact, we performed the analysis proposed by Templeton [39] for the total cladogram (Figure 5). As we move towards the higher-step clades, the average geographic pairwise distance remains different from zero; this happens even at the highest-clade step for six populations from five areas: Hondo Sur, Las Eras, Poza Sur, Poza Norte de Torreblanca, Balsa de Santed 1 and Estany d'en Túries. Therefore, secondary contact between two or more mtDNA clades at the highest level is the suggested inference for these ponds. In Hondo Sur, clades 3-1, 3-2 and 3-4 were found with frequencies 3.33%, 93.33% and 3.33%, respectively. In Las Eras, clades 3-2 and 3-4 overlapped with frequencies 90% and 10%, respectively. In Poza Sur, three clades (3-1, 3-2 and 3-3) overlapped with frequencies 83.33%, 13.89%, and 2.78%, respectively. In Poza Norte, clades 3-1 and 3-3 appeared with equal frequencies. In Balsa de Santed 1, clades 3-2 and 3-4 have frequencies 43.48% and 56.52%, respectively. Finally, in Estany d'en Túries, clades 3-2 and 3-3 have frequencies 14.29% and 85.71%, respectively. The distribution of clades and patterns of secondary contact between them in these populations is illustrated in Figure 6.

**Figure 7:**
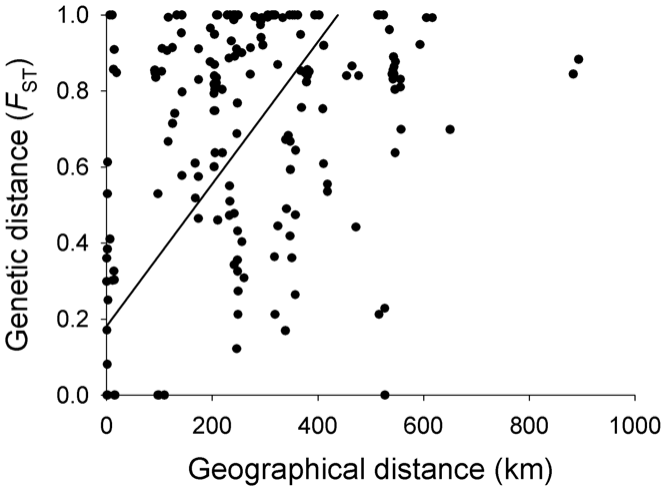
RMA regression for pairwise geographic distances and *F*_ST_ estimates. IBDWS detected a positive and significant correlation between genetic and geographical distance (genetic distance = 0.182+0.00187 geographical distance, in km).

*B. plicatilis* populations were highly subdivided (Figure 7; Table 3), with a global *F*_ST_ of 0.79 (*P*<0.001) and pairwise *F*_ST_ values ranging from −0.19 to 1. IBDWS detected a positive and significant correlation between genetic and geographical distance for the overall dataset (*r* = 0.37, *P* = 0.0009; genetic distance = 0.182+0.00187 geographical distance, in km).

**Table 3:** Pairwise *F*_ST_ estimates values (Tamura-Nei evolutionary model applied) between populations of *Brachionus plicatilis*.

	HOS	HOY	SA1	TOS	CHI	SAL	TUR	TON	GAL	ERA	SA2	CAP	MOJ	FUE	MAN	SLD	PET	CLO	ATA
HOY	**0.85**																		
SA1	**0.44**	**0.43**																	
TOS	**0.47**	**0.84**	**0.57**																
CHI	**0.68**	**0.94**	**0.67**	**0.71**															
SAL	**0.91**	**0.30**	**0.51**	**0.88**	**1.00**														
TUR	**0.70**	**0.89**	**0.61**	**0.67**	**0.92**	**0.96**													
TON	**0.55**	**0.87**	**0.46**	0.36	**0.91**	**0.97**	0.17												
GAL	**0.36**	**0.89**	**0.41**	**0.52**	**0.91**	**1.00**	**0.75**	**0.61**											
ERA	**0.44**	**0.86**	**0.40**	**0.55**	**0.76**	**0.95**	**0.70**	**0.54**	**0.31**										
SA2	**0.87**	**0.33**	**0.30**	**0.83**	**0.99**	**1.00**	**0.92**	**0.91**	**1.00**	**0.90**									
CAP	**0.85**	**0.26**	0.23	**0.81**	**0.99**	**1.00**	**0.88**	**0.83**	**1.00**	**0.87**	0.00								
MOJ	**0.84**	−0.07	0.12	**0.79**	**0.99**	0.86	**0.83**	0.60	0.99	**0.82**	0.69	0.47							
FUE	**0.85**	0.42	0.21	**0.80**	**0.99**	**1.00**	**0.84**	0.64	**1.00**	**0.84**	**1.00**	**1.00**	0.67						
MAN	**0.89**	**0.58**	**0.46**	**0.84**	**0.99**	**1.00**	**0.92**	**0.91**	**1.00**	**0.91**	**1.00**	**1.00**	**0.95**	**1.00**					
SLD	**0.86**	0.17	**0.36**	**0.82**	**0.98**	**0.91**	**0.85**	0.75	**0.95**	**0.84**	**0.77**	**0.64**	0.38	0.59	**0.80**				
PET	**0.85**	−0.04	**0.34**	**0.82**	**0.97**	0.30	**0.88**	**0.83**	**0.93**	**0.85**	**0.48**	**0.36**	−0.18	**0.49**	**0.74**	0.33			
CLO	**0.85**	0.00	0.21	**0.80**	**0.99**	0.00	**0.84**	0.64	**1.00**	**0.84**	**1.00**	**1.00**	0.00	1.00	**1.00**	0.53	−0.19		
ATA	**0.85**	0.08	0.27	**0.81**	**0.99**	0.00	**0.86**	0.75	**1.00**	**0.85**	**1.00**	**1.00**	0.25	1.00	**1.00**	0.61	−0.07	0.00	
CAS	**0.85**	0.00	0.21	**0.80**	**0.99**	0.00	**0.84**	0.64	**1.00**	**0.84**	**1.00**	**1.00**	0.00	1.00	**1.00**	0.53	−0.19	0.00	0.00

For population acronyms see Table 1. Values in bold indicate statistical significance (*P*<0.05).

## Discussion

The addition of new ponds in the present study has improved the understanding of phylogeographic patterns of *B. plicatilis* in the Iberian Peninsula. In support of previous findings [5], *B. plicatilis* populations were strongly subdivided and displayed a marked phylogeographic structure, but also showed evidence of secondary contacts. The two deep lineages (A and B) reported by Gómez et al. [5] were also found, although in the present study a larger level of spatial overlap was observed. Possibly, lineage A was in the area first and lineage B expanded later, in agreement with the lower genetic diversity of the latter.

### Phylogeography and population structure within clades

The cladogram showed some differences with respect to the phylogram, mainly due to the nesting rules in the nested clade phylogeographic analysis. The cladogram displayed four highest-step nested clades (3^rd^-step) while the phylogram displayed two main lineages (A and B). Lineage B corresponded to nested clade 3-4 in the cladogram, while clades 3-1 and 3-3 corresponded to well-supported branches from lineage A in the phylogram. Nested clade 3-2 grouped basal haplotypes and less supported branches in the phylogenetic tree, corresponding to its central position on the cladogram. The cladogram found here differs slightly from that in Gómez et al. [5] in that we found an extra 3^rd^-step clade (3-1 in this study), and a higher complexity in clade 3-2. These changes resulted from new haplotypes found mainly in Hondo Sur lagoon. Interestingly, haplotypes from this lagoon occupy a central position in the cladogram, which results in changes in the inference of the location of a putative refugium of lineage A in *B. plicatilis* (see below). Accordingly to NCPA, lineage A (clade 3-2) shows an isolation-by-distance pattern (but see below), with (1) contiguous range expansion along some lagoons of the coastal chain (clade 2-3, i.e., Estany d'en Túries, Poza Sur, and Hondo Sur), and (2) possibly range expansion in the northern inland ponds (clade 2-4, i.e. Laguna de las Eras, Balsa de Santed 1, Gallocanta, and Salada de Chiprana). The lower haplotype diversity of northern inland populations (clade 2-4) and the position of their haplotypes in the network as tips are findings consistent with an expansion from outside the area, probably from the coastal chain of lagoons. This conclusion differs from what was proposed by Gómez et al. [5], since they suggested that there was a past fragmentation event between inland and coastal ponds. Nevertheless, both explanations are compatible if an initial range expansion was followed by isolation between origin and expansion areas.

Although some haplotypes were more widespread (e.g. haplotype 21), the results point out a general pattern of private haplotypes, present in single lakes. Also, we can conclude that there are separated phylogeographic groups, one distributed along the coast and northern inland ponds and the other mainly in southern inland ponds and a few ponds from the North. This finding is in agreement with expectations of the monopolisation hypothesis (but see below). There are still some unsampled areas, mainly from the Guadiana and the Guadalquivir basins, which could affect our conclusions to some extent, probably for clade 3-4 (lineage B). In addition, samples from North Africa could help us in determining the route of colonization or even, possible glacial refugia in this area.

### Refugia and colonization pattern

Glacial refugia are defined as zones with suitable conditions for species to survive during the last Pleistocene glaciations. Iberian, Balkan-Greece and Italian Peninsulas have been recognized as major glacial refugia for European species [42]. In particular, the Iberian Peninsula has been acknowledged as a main glacial refugium, or more precisely as a complex of multiple glacial refugia, ‘refugia within refugia’ as expressed by Gómez and Lunt [12]. As it happens for other dispersal centres, glacial refugia are characterized by having larger allelic or haplotype richness than more recently colonized areas, even though zones of secondary contact of divergent lineages –contact zones– can show also high genetic diversity [42], [43].

In the case of *Brachionus* rotifers, areas within the Iberian Peninsula showing higher haplotype diversity within a clade are thus good candidates to be glacial refugia for the different lineages. Although much work is still needed in this area, there is some evidence of persistence of saline conditions in some Iberian lakes, during glacial advances [44], [45]. One of the main problems when trying to precisely establish glacial refugia for aquatic invertebrates in the Iberian Peninsula is the loss of their habitats due to human activities [7]. On one hand, many small salt ponds have been modified for agricultural purposes and, on the other, many coastal ponds are increasingly threatened by urbanization. Another important caveat in determining glacial refugia is rotifer capability for long-distance dispersal [46], which may confound patterns of regional colonization. Finally, even though we tried to retrieve samples from as many possible salt ponds in the Iberian Peninsula, the distribution of halophilic rotifers in the Iberian Peninsula as a whole is still incompletely known, and sampling can be affected by the patchy distribution of diapausing eggs in the sediment [47]. The apparent alternation between *B. plicatilis* and *B. manjavacas* often found on the superficial sediment of ponds (Montero-Pau et al., submitted) also makes the retrieval of the focus species in a particular sampling trip difficult. It should be taken into account that all these factors could be affecting the data when establishing the possible glacial refugia of *B. plicatilis*.

The average sequence divergence between the two main *B. plicatilis* lineages was 2.7%. Based on sequence divergence rates described for arthropods (1.4% of divergence per million years) [38], the split time between lineages A and B would be around 2 million years ago, predating the Pleistocene ice ages, which would mean that both lineages have survived at least several ice ages. Within lineage A, we found that coastal populations have higher haplotype diversity than inland populations. Within the coastal pond chain, the central position of Hondo Sur population in the cladogram suggests this area harboured the oldest glacial refugium for lineage A. Originally, Hondo Sur was part of a coastal lagoon, but due to silting and human activities its size was reduced through time [49]. The NCPA suggests that the species expanded from this refugium through the Iberian Mediterranean coast and entered inland regions through the Ebro basin.

Regarding lineage B, its low haplotype diversity and the close relationships of the haplotypes involved are consistent with a more recent arrival in the study area. Nonetheless, the geographic structuring of this lineage in the region and the occurrence of private haplotypes in Duero, Guadiana and Júcar-Segura basins suggest a recent glacial refugium in the Iberian Peninsula. As discussed above, more extensive sampling of the Guadiana and Guadalquivir basins and the southern coastal lagoons would be needed to conclude on the location of a putative glacial refugium for this lineage. A possibility would be that a refugium was in yet unsampled areas on the south of the Iberian Peninsula or North Africa.

From the glacial refugia, a pattern of serial founder effects resulting from successive colonizations from nearby habitats could explain the correlation between genetic and geographical distances in *B. plicatilis*. This idea was initially put forward for humans in their expansion throughout the world [50] and subsequently proposed to explain the correlation between genetic and geographical distances found in several continental aquatic invertebrates including *B. manjavacas* [6], the brine shrimp *Artemia salina* [7], an Australian isopod [51] and for *B. plicatilis* at a worldwide level [46]. Serial founder effects involve a single individual or a small group of individuals from nearby habitats colonizing the closest available habitat and this new population serving as origin for further colonizations, creating this pattern of correlation between genetic and geographical distances. Although we found a significant and positive correlation between these two distances, an important part of the variation in the genetic distance remains unexplained by geographic distance, pointing out that other factors affect this relationship. A few possible explanations could be: first, the occurrence of more than one refugia from which expansion happens; second, the patterns of secondary contact between genetically divergent lineages; third, the occurrence of long-distance dispersal; and fourth, the recent episodes of range expansions where single or highly related haplotypes have recently colonized an area. The results from the present study indicate that all of these factors would be at play in the *B. plicatilis* system. Despite all these factors the relationship between genetic and geographic distance was still significant.

### Phylogeographic concordance in the Iberian Peninsula

The phylogeographic patterns of *B. plicatilis* and *B. manjavacas* in the Iberian Peninsula show remarkable similarities [6]. First, both species must have been sympatric in the Iberian Peninsula for a long time. Both species show two main mtDNA lineages, and, interestingly, one of these lineages in each species had much higher genetical diversity than the other. Therefore, in both species there is a major lineage that seems to have survived during several ice ages in the Iberian Peninsula, and shows strong phylogenetic structure. By contrast, in both species a lineage with a recent expansion, which does not exclude colonization from outside the Iberian Peninsula, has been found. In both species, we found processes of range expansion, allopatric fragmentation, patterns of isolation-by-distance and secondary contact between lineages. Despite the localized and isolated character of the habitats of these rotifers (salt ponds and lakes), evidence for two glacial refugia for rotifers in the Iberian Peninsula (Guadiana basin and around El Hondo Nature Reserve area) was found. Each species might have survived in different basins, possibly varying in their salinity regime [6; Montero–Pau et al., submitted].

**Figure 8:**
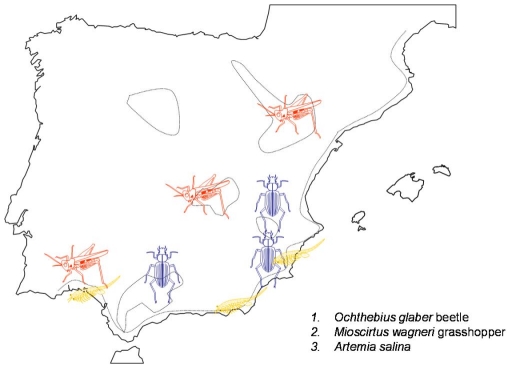
Map showing the possible glacial refugia of the invertebrates *Ochthebius glaber*, *Artemia salina*, *Mioscirtus wagneri* and the rotifers *B. plicatilis* and *B. manjavacas* in the Iberian Peninsula.

Recent studies on the phylogeography of halophilic invertebrates are indeed supporting a pattern of high population subdivision, regional endemism and multiple refugia within the Iberian Peninsula (Figure 8). Three Iberian refugia and patterns of serial colonization have been proposed for the brine shrimp *Artemia salina*. The hypothesized refugia would have been located around the southeastern area (Almería), the Doñana area and possibly a third refugium from which the East coast of the Iberian Peninsula and the Balearic Islands were colonized [7]. The endangered water beetle *Ochthebius glaber*, which lives in Iberian hypersaline streams, has been found in three groups of populations: in the basins of the Segura, Guadalquivir and Júcar rivers [14]. The authors found signatures of range expansion, past fragmentation and restricted gene flow with isolation by distance. In the halophylic grasshopper *Mioscirtus wagneri*, certain similarities are apparent with the patterns found for *Brachionus* rotifers [15], since there are three phylogenetic groups geographically isolated among them and located in north-east, central–south-east and south-west Iberia. Genetic and geographical distances are also correlated in these grasshoppers. The authors suggested the pattern resulted from vicariance of a single ancient, more widespread population in the Iberian Peninsula, which contracted with the retraction of halophylic environments, possibly in the Early Pleistocene. The global picture arising from these studies and our results is that southern Iberian Peninsula harboured glacial refugia for different halophilic organisms (Figure 8). Nevertheless, we must compare the rotifers' phylogeographic patterns with those of the beetle and the grasshopper with caution, since long distance dispersal seems more likely in rotifers. For this reason, in the case of rotifers the presence of North African refugia is plausible, as crossing of the Gibraltar straits is a more likely scenario.

### Secondary contact: Implications for the Monopolization hypothesis

One prediction of the Monopolization hypothesis [4] is that events of secondary contact are unlikely. The first migrants form a resident population which will tend to monopolize the habitat quite quickly, hampering the later effective colonization of other migrants. This monopolization is assumed to be mainly due to two facts: fast growth population rates (a numerical effect) and the rapid adaptation to the new habitat (local adaptation effect). Indeed, many aquatic organisms show little signs of secondary contact, with the different lineages being allopatric, but both *Brachionus* species analyzed so far do show signs of overlap in space between lineages. There are several, non-exclusive factors that could explain this finding.

First, the monopolization applies to populations of the same species and thus, one possibility is that the different lineages found were in fact different species, occupying differentiated niches. However, in our case, this is not supported by population genetic studies with nuclear loci, such as microsatellites [52], [53], which shows Hardy Weinberg equilibrium, and so recombination, in ponds where different mitochondrial lineages co-occur. Moreover, no signs of reproductive isolation between clones belonging to the two mitochondrial phylogenetic lineages A and B were found [18], supporting that these two lineages effectively belong to a single biological species.

Second, Iberian saline ponds and lakes are known for being highly variable, with strong fluctuations in water level and environmental conditions [43]. Assuming that local adaptation is common, but also that rotifer habitats have very variable ecological conditions, an immigrant arriving to a pond where conditions have just changed could have opportunities to thrive when its source pond would have similar conditions to the new conditions in the target pond, even if this pond would have already a well-established population. We hypothesize that this could increase the chances of secondary contact between lineages evolved in allopatry. Interestingly, these rotifers disperse through diapausing eggs, a resistant form that can survive for decades in the sediment [47], until environmental conditions are favourable to hatch. A previous study with rotifers in the Iberian Peninsula [53] suggested local adaptation and ecological specialization in some Iberian populations of this rotifer, but the authors stressed that environmental fluctuation seemed to hamper to some extent local adaptation to salinity and temperature in most populations.

Third, divergent lineages could have arrived to the habitat almost simultaneously, effectively establishing a population at the same time and before either of them could effectively monopolize the new habitat.

Finally, as suggested in other studies, hybrid vigour could help explain the pattern observed since inbreeding depression could affect the initial phases of colonization of a new habitat [54]. If a few migrants from the same origin colonize a new habitat, they will probably be affected by inbreeding depression [54]. Migrants belonging to other lineage and arriving later could mate with them, giving place to offspring with hybrid vigour [55].

### Conclusions

Some of our results are consistent with previous studies [5]: first, *B. plicatilis* is made of two main phylogeographic lineages in the Iberian Peninsula, with lineage A being genetically more diverse than lineage B; second, lineages survived for several ice ages and show strong phylogeographic structure; and third, the genetically much more diverse and structured lineage A is likely the oldest one, possibly surviving several climatic cycles in the area, while lineage B expanded its range later. In contrast, additional samples included in this study have revealed that a population not sampled in previous studies –Hondo Sur– played a key role in the phylogeography of *B. plicatilis*. The central position of Hondo Sur population in the cladogram suggests that this area harboured the oldest glacial refugium for lineage A. Moreover, in this study we have found processes of allopatric fragmentation and range expansion with patterns of isolation-by-distance in *B. plicatilis*. Historical events (serial colonizations) could explain the isolation-by-distance pattern. The study also confirms that secondary contact is more frequent in these rotifers than previously thought. Finally, phylogeographic patterns shown by *B. plicatilis* in the Iberian Peninsula are highly concordant with those found in *B. manjavacas* and have certain similarities with other organisms dwelling in halophylic environments.

## Supporting Information

Figure S1 **Relationship between sample size (number of individuals sequenced) and the number of haplotypes.**
(TIF)
