# Supplementary figures and images for: Widespread Secondary Contact and New Glacial Refugia in the Halophilic Rotifer Brachionus plicatilis in the Iberian Peninsula
Source: PLoS One. 2011 Jun 16;6(6):e20986. doi: 10.1371/journal.pone.0020986 (PMC3116854; doi:10.1371/journal.pone.0020986)

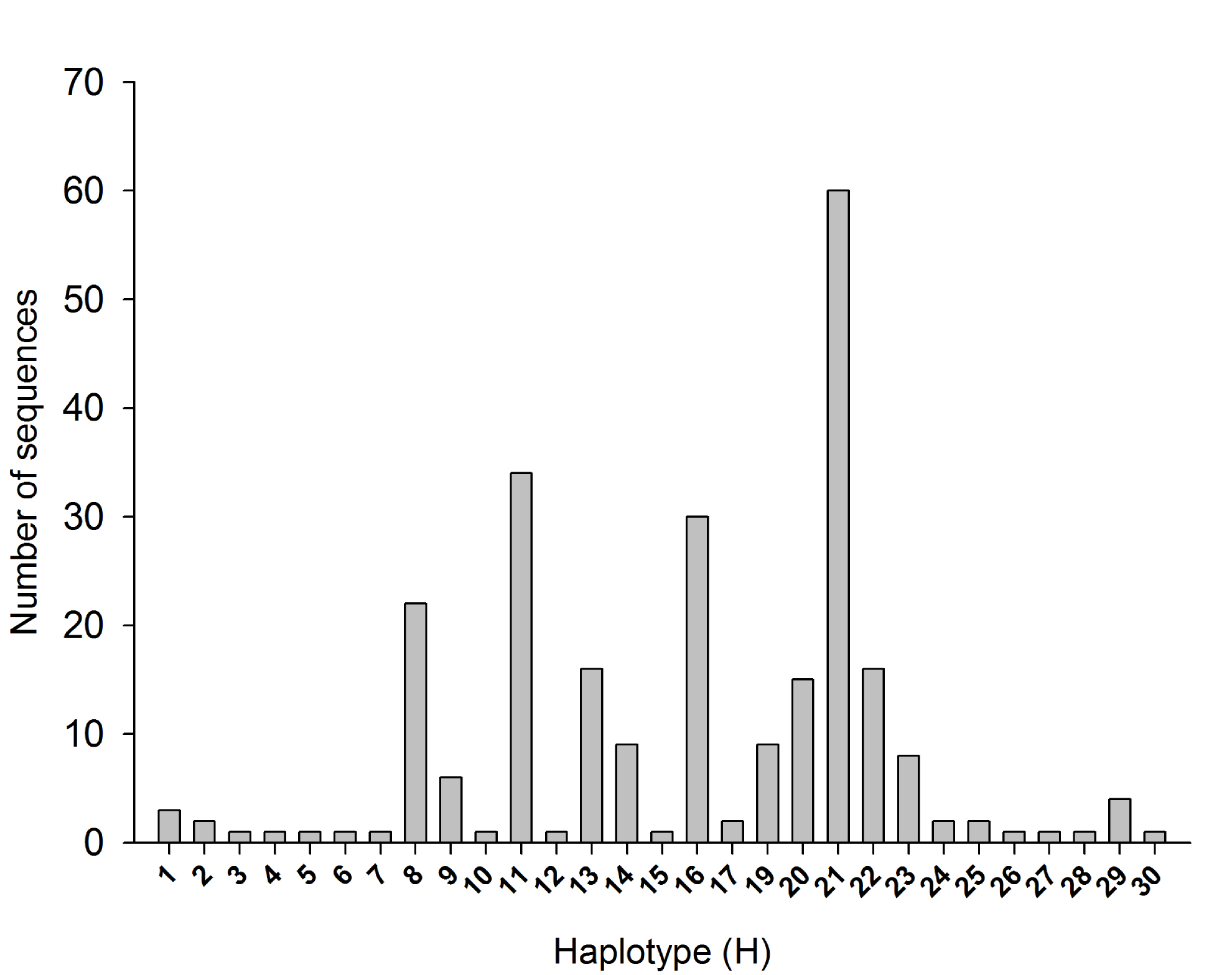

Supplement: Figure S1 — Relationship between sample size (number of individuals sequenced) and the number of haplotypes. (TIF) [file pone.0020986.s001.tif]
